# Aldo-Keto Reductase 1B10 (AKR1B10) Restrains Trophoblast Cell Invasion via the ERK Signaling Pathway

**DOI:** 10.3390/ijms27177728

**Published:** 2026-08-28

**Authors:** Suohao Peng, Jiaxuan Cai, Xiaohua Lei, Chen Huang

**Affiliations:** 1 Department of Biomedical Engineering, Southern University of Science and Technology, Shenzhen 518055, China; 2 Faculty of Pharmaceutical Sciences, Shenzhen University of Advanced Technology, Shenzhen 518107, China; 3 Center for Energy Metabolism and Reproduction, Institute of Biomedicine and Biotechnology, Shenzhen Institutes of Advanced Technology, Chinese Academy of Sciences, Shenzhen 518055, China

**Keywords:** AKR1B10, placenta, trophoblast invasion, ERK signaling pathway

## Abstract

Trophoblast invasion is crucial for the establishment of a functional placenta. Defects in this process can cause adverse pregnancy outcomes, such as miscarriage and preeclampsia. Aldo-keto reductase family 1 member B10 (AKR1B10), a key cytoplasmic oxidoreductase that converts retinoids, retinaldehyde isoprenoids, and lipid peroxidation-derived reactive aldehydes into their corresponding alcohols, regulates cell proliferation, inflammation, and metastasis in cancers. Here, our results demonstrated that AKR1B10 is highly expressed in the ectoplacental cone (EPC) of the mouse placenta. Deficiency of AKR1B10 leads to excessive trophoblast giant cell (TGC) invasion, impaired trophoblast cell differentiation of the junctional zone, and accelerated maternal spiral artery remodeling. Mechanistically, loss of AKR1B10 suppressed extracellular regulated protein kinase (ERK) phosphorylation in both murine placentas and human HTR8/SVneo cells, further promoting HTR8 cell migration and invasion in vitro. Our findings identify AKR1B10 as a critical regulator that restrains trophoblast invasion in the placenta, suggesting it as a new therapeutic target for placental disorders.

## 1. Introduction

Aldo-keto reductase family 1 member (AKR1B10) is a monomeric, soluble, NADPH-dependent oxidoreductase belonging to the AKE1B subfamily. It has been defined as a new cytosolic enzyme that converts retinoids, retinaldehyde isoprenoids, and lipid peroxidation-derived reactive aldehydes to their corresponding alcohol [1]. This reaction is thought to be involved in cell proliferation [2,3], metastasis [4], and drug resistance [5] in cancer cells. Indeed, AKR1B10 has been considered a potential diagnostic and therapeutic biomarker for multiple types of cancer, including hepatocellular carcinoma (HCC) [6], esophageal squamous cell carcinoma (ESCC) [7], gastric cancer (GC), and colorectal cancer [8]. In the mechanism, AKR1B10 sustains the mitogen-activated protein kinase (MAPK)/extracellular signal-regulated protein kinase (ERK) pathway and acetyl-CoA carboxylase 1 (encoded by ACACA) activity [9,10,11] to regulate cell viability, inflammation [12,13], migration, and cancer metastatic progression [14]. Recently, AKR1B10 was reported to be expressed in the placenta and trophoblast cells and to regulate AMPK signaling and mitochondrial ATP production in trophoblasts [15,16], pointing to a potential role for AKR1B10 in placental development.

Placental development and function are critical for the success of pregnancy and maternal and fetal health. In placental development, the trophoblast derives from the extraembryonic tissue in the outer layer, trophectoderm (TE), of the embryo at the blastocyst stage. After embryo implantation, the mural TE that is not in direct contact with the inner cell mass ceases dividing and differentiates into primary trophoblast giant cells. The polar trophectoderm that overlies the inner cell mass continues to proliferate and gives rise to all trophoblast cell types of the placenta. The polar trophectoderm gradually develops into extraembryonic ectoderm (ExE), chorionic plate, and the ectoplacental cone (EPC) [17], which give rise to the spongiotrophoblast layer (junctional zone in placenta) and the trophoblast giant cells (TGC) [18]. These TGCs have similar activities to extravillous cytotrophoblast cells (EVT) in humans, penetrate the basement membrane of the maternal endometrium, invade the decidua and myometrium, remodel maternal spiral arteries, and secrete hormones for proper pregnancy adaptation [19]. Failures in this process are associated with the occurrence of different gestational diseases, including preeclampsia [20], miscarriage [21], and fetal growth restriction [22]. The extracellular regulated protein kinase (ERK) signaling pathway, which is a classic regulatory factor for cell proliferation [23], migration, and differentiation [24,25,26], participates in trophoblast lineage development, chorioallantois branching, and labyrinth development [27]. Mutants of the ERK/MAP kinase gene reduced the vascularization of the labyrinth, but they promoted the excessive accumulation of TGCs in the labyrinth [28,29]. A recent study using human trophoblast single-cell analysis suggested that MAPK signaling is significantly enriched in the transition of cytotrophoblasts to EVT branches, and inhibited MAPK signaling induces HLA-G expression in the differentiation of human trophoblast stem cells (hTSC) [30].

In this study, we investigated AKR1B10 expression and its downstream signaling pathway during mouse placental development. Using AKR1B10 knockout mice and an immortalized human trophoblast cell line (HTR8/SVneo), we demonstrated that AKR1B10 deficiency promotes the invasion of TGCs and maternal spiral artery remodeling, impairing the differentiation of spongiotrophoblast cells and glycogen trophoblast. Knockdown of AKR1B10 in trophoblasts promoted HTR8 cell migration and invasion and suppressed ERK phosphorylation in both murine placentas and human HTR8/SVneo cells. This study demonstrated that AKR1B10 is a critical regulator of trophoblast invasion in the placenta, giving new insights into AKR1B10 and ERK signaling in trophoblast invasion and placental function.

## 2. Results

### 2.1. The Expression of AKR1B10 During Murine Placental Development

**Figure 1:**
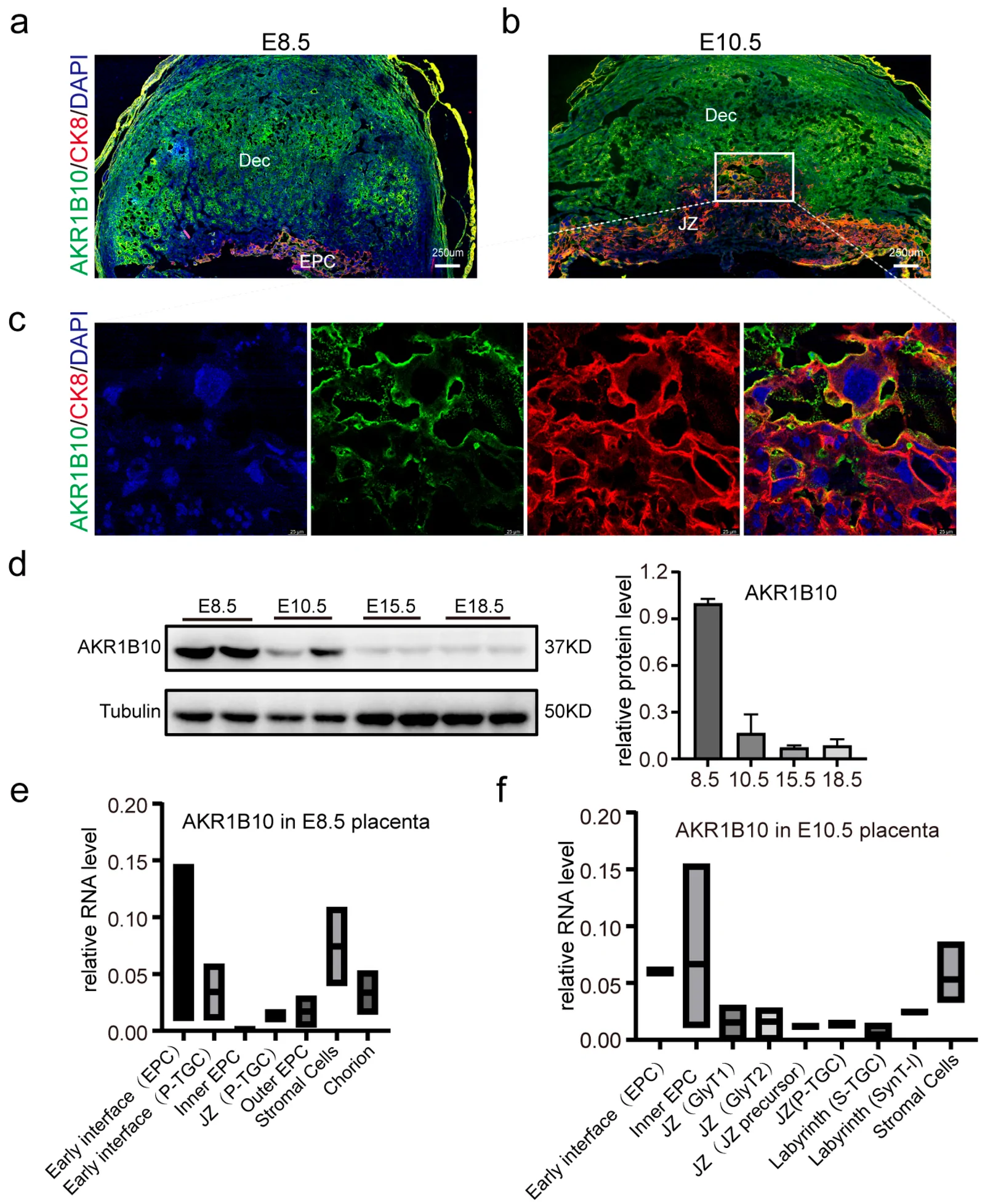
**The active expression of AKR1B10 in placental development**. (**a**–**c**) AKR1B10 expression (green) was examined at E8.5 (**a**) and 10.5 (**b**,**c**) placentas containing trophoblast marker cytokeratin 8 CK8 (red) and DAPI-stained nuclei (blue). Representative staining images at E8.5 of the placenta showed that the AKR1B10 signal accumulated in the primary trophoblast giant cells of the ectoplacental cone (EPC) and in the stromal cells of the decidua (Dec). (**b**) At E10.5, AKR1B10 was expressed in the decidual stromal cells and junctional zone (JZ) trophoblast giant cells. The dashed square in (**b**) indicates the region shown at higher magnification in (**c**). (**c**) The square-frame image in E10.5 placentas further showed that the AKR1B10 signal was accumulated on the trophoblast giant cell (TGC) in the invasive placental junction zone. (**d**) Expression of AKR1B10 was examined and quantified on different days (E8.5, 10.5, 15.5, and 18.5) of the mouse placenta. Each group has two placentas from two mice. (**e**,**f**) The boxplots show the mean AKR1B10 expression in E8.5 and E10.5 placental sections from the publicly available spatiotemporal transcriptomic data (*n* = 2) [31]. EPC (ectoplacental cone), P-TGC (primary trophoblast giant cell), JZ (junctional zone), and GlyT (glycogen trophoblast).

To investigate the role of AKR1B10 during pregnancy, we first examined its expression and localization in the mouse placenta. Immunostaining revealed that AKR1B10 is primarily expressed in decidual stromal cells and extraembryonic trophoblasts of EPC (Figure 1a). As placental development progressed, AKR1B10 expression was detected in the placental decidua and junctional zone in E10.5, containing trophoblast cell marker CK8 (Figure 1b). Higher-magnification images at E10.5 showed that AKR1B10 accumulates in parietal trophoblast giant cells of the junctional zone, which deeply invade into decidual tissue (Figure 1c). Placental protein expression indicated that AKR1B10 had high expression at E8.5 and E10.5 but decreased at E15.5 and E18.5 (Figure 1d). Consistent with this, analyses of published spatiotemporal transcriptomic data confirmed that higher AKR1B10 expression was observed in the TGCs of EPC and stromal cells of decidua between E8.5 and E10.5 placenta [31] (Figure 1e), but lower expression was observed in differentiated EPC-derived trophoblast lineages, including glycogen trophoblasts (GlyTs) and junctional zone trophoblast precursors (JZ precursor) (Figure 1f). Together, these results suggest that AKR1B10 is predominantly expressed during early placental development and may play a role in EPC-derived trophoblast function.

### 2.2. Loss of AKR1B10 Contributes to Aberrant Trophoblast Invasion in Mouse Placenta

**Figure 2:**
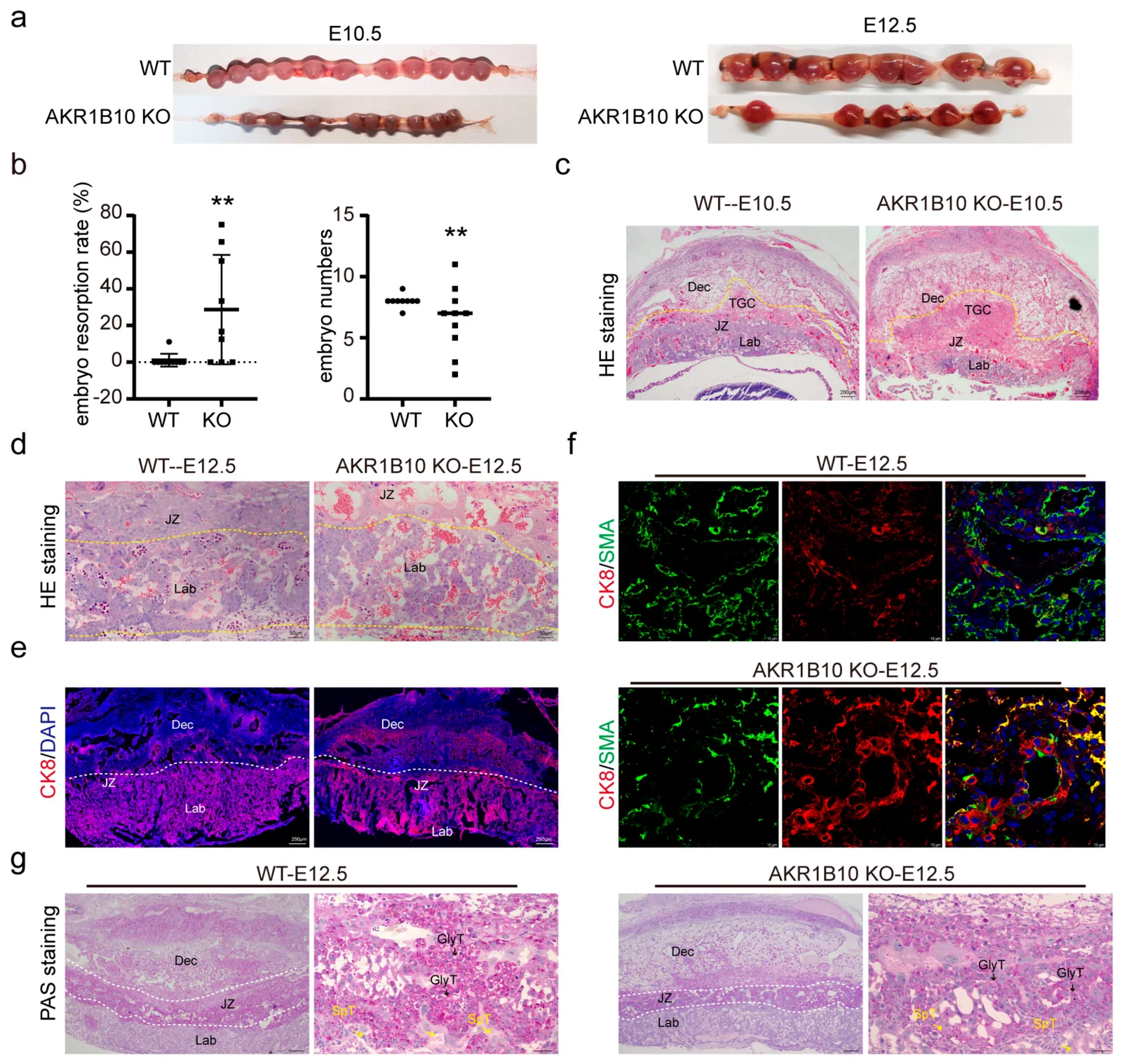
**Deficiency of AKR1B10 results in aberrant trophoblast invasion.** (**a**) The phenotypes of embryos in E10.5 and E12.5 uteri from C57BL/6 (WT) and AKR1B10 knockout (KO) mice. (**b**) Resorption rate and embryo numbers in E12.5 from WT and KO mice. *n* = 10 for WT mice, and *n* = 8 for AKR1B10 KO mice. All graphs depict mean (SD) values with a non-parametric Mann–Whitney U test. ** represents *p* < 0.01. (**c**,**d**) Representative image of WT and AKR1B10 KO placenta at E10.5 and E12.5. The yellow line indicates the boundary of the placental invasive trophoblast. (**e**) The staining of the placenta from WT and AKR1B10 knockout mice at E12.5 with CK8 staining. The white line indicates the interface between maternal decidual tissue and the placenta junctional zone tissue. Labyrinthine (Lab), junctional zone (Jz), and decidua (Dec). (**f**) Representative images showed remodeling of spiral arteries in WT and AKR1B10 KO placentas at E12.5, stained with CK8 (trophoblast) and α-smooth muscle actin (α-SMA). (**g**) The image of PAS staining on the WT and AKR1B10 KO placenta at E12.5. Scale bar = 200 μm. The white line indicates the layer of the placental junctional zone (Jz). The smaller version shows the distribution of glycogen trophoblasts (GlyT, black arrows) and spongiotrophoblasts (SpT, yellow arrows) in the junctional zone. Scale bar = 50 μm.

To investigate the function of AKR1B10 in placental development, we established a global knockout (KO) mouse by CRISPR-Cas9-mediated deletion of exons 2 to 9. Phenotype analysis showed that AKR1B10 deficiency led to a considerable decrease in litter size (Figure 2a) and increased incidence of embryonic resorption during gestation (Figure 2b,c). However, no overt differences in maternal body weight or general morphology were observed between KO and wild-type mice.

Histological examination of placentas by H&E staining revealed that a large number of TGCs had invaded into the decidua of WT mice at E10.5 (Figure 2d). These invasive TGCs were immunolabeled with CK8 in E12.5 WT placentas and mainly accumulated around maternal spiral arteries (Figure 2e), and they displaced the vascular smooth muscle cells marked with α-smooth muscle actin (α-SMA, green) to remodel the maternal spiral arteries (Figure 2f). In contrast, extensive invasion of TGCs was observed in E10.5 AKR1B10 KO placentas, with TGCs migrating throughout all decidual tissue in E12.5 KO placentas (Figure 2e), and this facilitated the process of artery remodeling, with a large number of vascular smooth muscle cells disappearing in the E12.5 AKR1B10 KO placenta (Figure 2e). H&E staining in the placenta further showed that the facilitated artery remodeling promoted maternal blood cell perfusion in the labyrinth and junctional zone tissue in AKR1B10 KO placentas (Figure 2d). The spongiotrophoblast (SpT) and trophoblast glycogen cell (GlyT), which are also derived from the EPC, were significantly decreased in E12.5 AKR1B10 KO placentas when examined with periodic acid-Schiff (PAS) staining (Figure 2g). These observations suggest that AKR1B10 deficiency facilitated trophoblast giant cell invasion and maternal artery remodeling but impaired trophoblast differentiation in the junctional zone.

### 2.3. The ERK Signaling Pathway Was Dysregulated in AK1B10 KO Placenta

**Figure 3:**
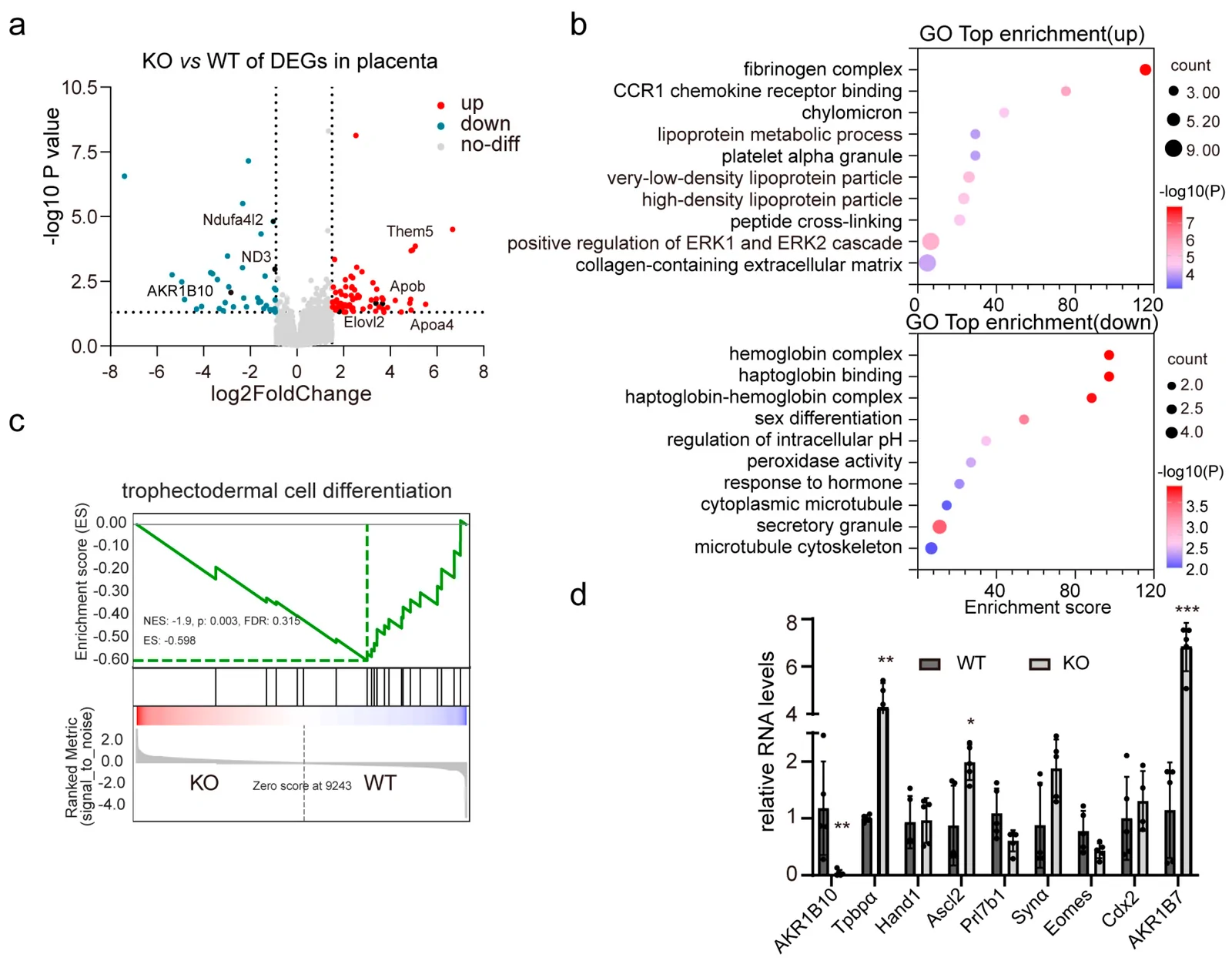
**Loss of AKR1B10 results in dysregulated lipid metabolism and the ERK pathway in the placenta.** (**a**) Volcano plot shows the differentially expressed genes (DEGs) in the E10.5 placenta of wild-type (WT) mice (*n* = 3) and AKR1B10 knockout (KO) mice (*n* = 3). Dots represent significantly downregulated transcripts with a *p*-value of ≤0.05 and 1.5 ≤ log2-fold change of ≤−1.5. (**b**) The bar graph shows the top upregulated and downregulated pathways from significantly changed genes in the AKR1B10 KO placenta. (**c**) GSEA analysis showed that the pathway of trophectodermal cell differentiation was significantly decreased in the AKR1B10 KO placenta. (**d**) qRT-PCR validates the expression of TSC/EXE-sustaining genes (*Cdx2*, *Eomes*); differentiated trophoblasts, including SpT and TGC (*Tpbpα*, *Hand1*, and *Ascl2*); GlyT (*Pr17b1*); syncytiotrophoblast (*Synα*)-specific transcripts; and *AKR1B10* and *AKR1B7* (two members of the aldo-keto reductase family 1) from WT (*n* = 5) and AKR1B10 KO (*n* = 5) placentas. * *p* < 0.05; ** *p* < 0.01, *** *p* < 0.001 showed the comparison of RNA level between WT and KO placenta. This assay was repeated three times and analyzed with a non-paired and two-tailed Welch’s *t*-test and Mann–Whitney U test.

To identify the dysregulated pathways in AKR1B10 KO placentas, we performed RNA sequencing on WT and KO placental tissue in E10.5. AKR1B10 deficiency altered the expression of 182 transcripts, with 51 downregulated and 131 upregulated (false detection rate-corrected *p* < 0.05, |log2FC| > 1.5; Figure 3a and differentially expressed genes in Supplementary File). Gene Ontology (GO) analysis suggested that the lipid metabolism-related pathway “lipoprotein metabolic process” was upregulated in the AKR1B10 KO placenta. Similarly, the pathway “regulation of ERK1 and ERK2 cascade” was also upregulated in KO placentas (Figure 3b). Gene set enrichment analysis (GSEA) suggested that the genes related to “trophectodermal cell differentiation”, such as *Eomes*, which is involved in sustaining TSCs/EXE, was downregulated in the AKR1B10 KO placenta (Figure 3c). In contrast, the genes related to EPC trophoblast differentiation, like *Tpbpα*, *Ascl2*, and *Synα*, were all upregulated in the AKR1B10 placenta, suggesting that deficiency of AKR1B10 disrupts EPC-derived trophoblast differentiation. In addition, another member of the aldo-keto reductase family 1, *AKR1B7*, exhibited markedly elevated expression in KO mice, suggesting that AKR1B10-deficient mice may induce AKR1B7 expression in the placenta to compensate for impaired AKR1B10 function.

**Figure 4:**
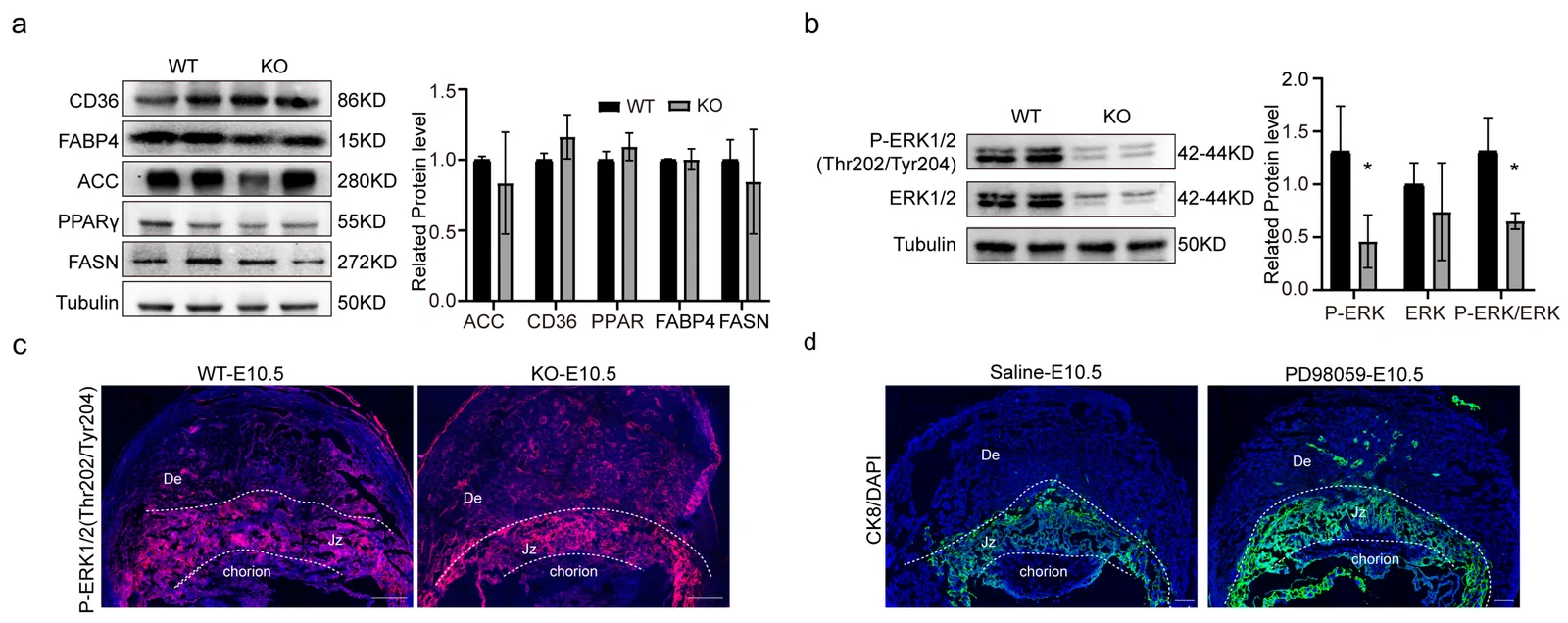
**Loss of AKR1B10 suppressed the phosphorylation of ERK in mouse placenta.** (**a**) The expression of lipid metabolism enzymes, including ACC, CD36, and PPARγ, in WT and AKR1B10 KO placentas was measured, and the relative protein levels were calculated three times. The graphs depict mean (SD) values and analysis with a non-paired and two-tailed Welch’s *t*-test. (**b**) The expression of P-ERK1/2(Thr202/Tyr204) and total ERK was measured in WT and AKR1B10 KO placenta, and the relative protein levels were calculated. The graphs depict mean (SD) values and analyses with a non-paired and two-tailed Welch’s *t*-test, * *p* < 0.05 represented the comparison of related protein level with WT and KO group. (**c**) The staining of the placenta from WT and AKR1B10 knockout mice at E10.5 with P-ERK1/2(Thr202/Tyr204) staining (red). The white line indicated the interface of maternal decidual tissue and the placenta junctional zone tissue. Junctional zone (Jz) and decidua (Dec). Bar = 500 μm. (**d**) E10.5 placentas from saline- and inhibitor-treated groups (PD98059, 6 mg/kg) were examined with CK8 (green), which indicated the effect of PD98059 on TGC invasion into maternal decidua tissue. Bar = 250 μm.

Further, we detected the signaling pathway associated with AKR1B10. Lipid metabolism-related genes, including FABP4 and CD36 (lipid-transport proteins), ACC and FASN (key enzymes for fatty-acid synthesis), and PPARγ (lipid metabolism regulator), were all not significantly changed between the WT and AKR1B10 KO placentas (Figure 4a). In contrast, the ERK/MAPK signal cascade was disrupted in the AKR1B10 KO placenta, as the phosphorylation of ERK1/2 at the Thr202/Tyr204 site was suppressed in the AKR1B10 KO placenta (Figure 4b). Immunostaining in E10.5 WT and KO placentas showed that the signal of P-ERK was enriched in the junctional zone and decidua tissue (Figure 4c). Therefore, we further treated WT pregnant mice with the ERK signaling inhibitor PD98059 (6 mg/kg, intraperitoneal injection) at E9.5; the placentas at E10.5 were collected and examined with CK8 staining. The results showed that inhibited ERK signaling indeed facilitated more TGCs invading into decidual tissue (Figure 4c).

### 2.4. Knockdown of AKR1B10 Promoted Cell Invasion and Suppressed ERK Phosphorylation in HTR-8/SVneo Cell Line

**Figure 5:**
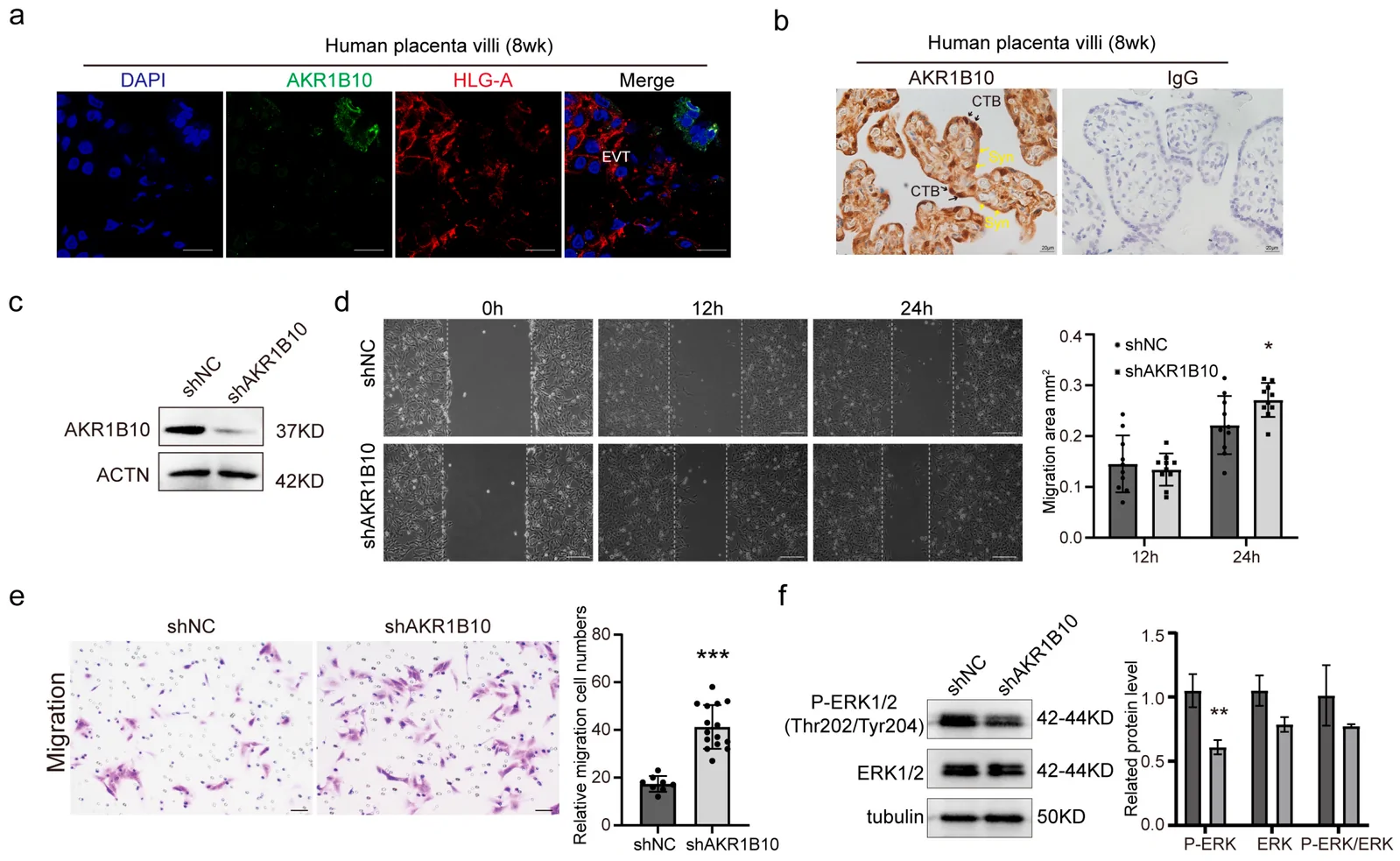
**Loss of AKR1B10 increased human trophoblast invasion and suppressed the ERK signaling pathway**. (**a**) Representative image of AKR1B10 expression in human placental villi at 8 wk; HLA-G is a marker of EVT. The merged image indicated that AKR1B10 shows little expression in cells expressing the EVT marker HLA-G. The scale bar in the images was 25 μm. (**b**) Representative image of AKR1B10 in human placenta villi at 8 wk. The image showed that AKR1B10 was mainly expressed in the cytotrophoblast (CTB, black arrow) but was also present on the syncytiotrophoblast (Syn, yellow arrows). (**c**) AKR1B10 expression was detected in shAKR1B10 and scramble (shNC) HTR-8 cell lines. (**d**) The wound-healing assay was performed at 0, 12, and 24 h using the shNC and shAKR1B10 HTR-8/SVneo cell lines. After 12 h of treatment, healing distances were measured to calculate the migration area. The scale bar in the images was 200 μm. Data are the mean (SD) of *n* = 9, representing triplicates of 3 different experiments with two-tailed Welch’s *t*-test, * *p* < 0.05 represented the comparison of migration area between shNC and shAKR1B10 group. (**e**) Assessment of the invasion potential of shAKR1B10 and scramble (shNC) in HTR-8 cell lines using the trans-well assay. After 24 h of culture, the migrated cells were counted to determine migration activity. The scale bar was 50 μm. Data are the mean (SD) of *n* = 8, representing triplicates of 3 different experiments with two-tailed Welch’s *t*-test, *** *p* < 0.001 represented the comparison of cell numbers between shNC and shAKR1B10 cell line. (**f**) The proteins of the P-ERK1/2(Thr202/Tyr204) and total ERK were measured in shAKR1B10 and shNC in HTR-8 cell lines, and the relative protein level was calculated. The graphs depict mean (SD) values and analysis with a two-tailed Welch’s *t*-test. ** *p* < 0.01 represented the comparison of related protein level between shNC and shAKR1B10 cell line from three repeated experiments.

In the human placenta, EVT perform a similar function as TGCs in mice, both of which invade the maternal decidua and uterine artery and determine arterial lumen remodeling. Here, we performed immunofluorescent staining to examine AKR1B10’s expression in the human placenta. The results showed that AKR1B10 is expressed in human placental cytotrophoblasts (8 weeks), but little accumulates in HLG-A-positive EVT cells and syncytiotrophoblasts (Syn) (Figure 5a,b). To further investigate the role of AKR1B10 in human trophoblasts, we knocked down AKR1B10 expression in a well-established invasive extravillous trophoblast cell line (HTR-8/SVneo) using lentiviral-delivered short hairpin RNAs (shAKR1B10) (Figure 5c). Knockdown of AKR1B10 indeed enhances trophoblast migration in a wound-healing assay (Figure 5d) and invasion in a trans-well assay (Figure 5e). Further, we detected ERK signaling pathway expression in HTR-8/SVneo cells. The results showed that knockdown of AKR1B10 suppresses the phosphorylation of the Thr202/Tyr204 site of ERK expression in trophoblasts (Figure 5f). These results suggest that AKR1B10 expression is closely associated with cell invasion and the ERK pathway in human trophoblasts.

## 3. Discussion

**Figure 6:**
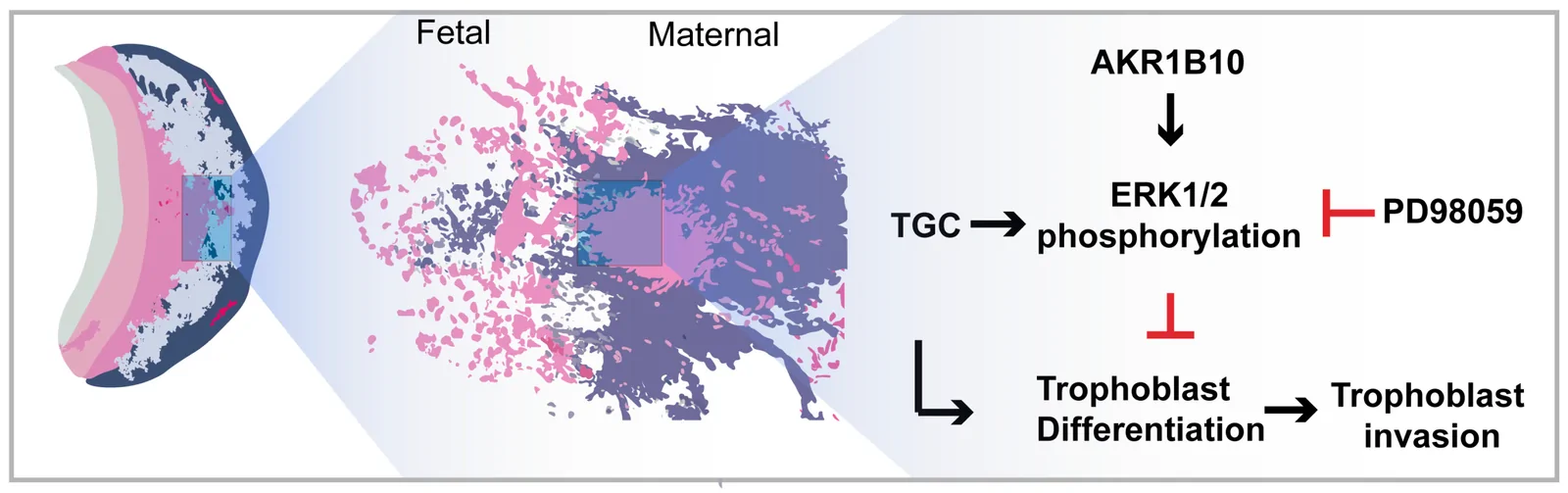
**Schematic representation of the physiological role of AKRB10 in placental trophoblast invasion.** This graphic depicts the role of AKR1B10 in regulating trophoblast differentiation and invasion through the ERK signaling pathway. During placental development, AKR1B10-mediated phosphorylation of the ERK signal regulated trophoblast cell differentiation in the ectoplacental cone (EPC). Deficiency of AKR1B10 or treatment with the inhibitor PD98059 suppressed the phosphorylation of the ERK signal, which impaired trophoblast cell differentiation in the junctional zone and accelerated trophoblast cell invasion.

Impaired trophoblast invasion is the primary cause of obstetric syndromes such as preeclampsia, fetal growth restriction, and unexplained stillbirth [25]. In this study, we identified AKR1B10 as a highly expressed factor in the mouse ectoplacental cone. Genetic ablation of AKR1B10 in mice significantly enhanced trophoblast giant cell invasion and maternal spiral artery remodeling, and impaired the differentiation of spongiotrophoblast cells and glycogen trophoblasts in the junctional zone. Mechanistically, loss of AKR1B10 suppressed ERK phosphorylation in both murine placentas and human HTR8/SVneo cells, promoting HTR8 cell migration and invasion in vitro. Further, we also showed that inhibited ERK signaling pathways in the placenta were highly associated with trophoblast giant cell invasion in the mouse placenta (Figure 6). These data suggest AKR1B10 as a regulator of trophoblast cell invasion and differentiation and offer a new therapeutic target for placental disorders.

Previous studies have suggested that ERK/MAP kinase participates in trophoblast lineage development, chorioallantois branching, and labyrinth development [27]. More noticeably, ERK1/2 MAP kinases are the downstream effectors of FGF receptor signaling, which is required for the maintenance of trophoblast proliferation in the extraembryonic ectoderm and ectoplacental cone [32]. FGF4 signaling in the extraembryonic ectoderm suppresses TGC formation, and its withdrawal results in the rapid differentiation of trophoblast stem cells into TGCs [33,34]. Here, our work in the mouse placenta implied that AKR1B10 participates in ectoplacental-cone-derived trophoblast cell invasions through the suppression of phosphorylated ERK activity. Inhibition of the ERK signaling pathway in the E9.5 placenta with PD98059 promoted trophoblast giant cell invasion in the E10.5 placenta. In human study, our results also confirmed that knockdown of AKR1B10 has an effect on cell invasion and the phosphorylation of ERK in the HTR8/SVNeo cell line. In future studies, more experiments should be performed on human trophoblast organoids and trophoblast stem cells to investigate AKR1B10 and the ERK pathway in human trophoblast differentiation.

The activity of retinoid oxidoreductive conversions in AKR1B10 is strongly associated with retinoic acid (RA) elimination during cancer development [35,36]. RA signaling has been considered a critical determinant of peripheral EPC cell fate, leading to the generation of trophoblast giant cells in the region facing uterine decidual tissues at the expense of spongiotrophoblast differentiation. TS cells cultured with exogenous retinoic acid were induced to form giant cells, and administration of RA during pregnancy resulted in an overabundance of giant cells [37]. Hence, we propose that AKR1B10 activity as a retinoid oxidoreductase in the placenta is another mechanism to regulate trophoblast invasion.

In humans, AKR1B10 was highly expressed in most cancer tissues and the epithelial cells of the small intestine, colon [38], and lung airway [39,40], but its expression was lower in normal tissues. Studies in transcriptomic and circulating proteomic analyses of non-alcoholic fatty liver disease (NAFLD) showed that AKR1B10 was highly expressed in the liver in the fibrosing steatohepatitis stage of NAFLD, suggesting AKR1B10 as a signature gene of fibrosis and steatosis [41,42]. The inhibitor of AKR1B10, sulindac, has an effect on the treatment of a non-alcoholic steatohepatitis (NASH) mouse model, showing a dramatic reduction in hepatic steatosis, plasma ALT levels, hepatic inflammation, and fibrosis [43]. This study suggested that AKR1B10 has an effect on placental development and offered a potential therapeutic target for the diseases of preeclampsia, miscarriage, and fetal growth restriction. In future studies, AKR1B10 inhibitors with a delivery tool in the placenta would be a promising strategy for the treatment of pregnancy disorders and to avoid embryo loss induced by gene ablation.

In summary, this study demonstrated that AKR1B10 has an effect on the trophoblast invasion of human and mouse placenta, offering insights into placental disorders and potential therapeutic targets.

## 4. Materials and Methods

### 4.1. Animals’ Care and Treatment

The animal experiment procedures in this study were reviewed and approved by the Institutional Animal Care and Use Committees (IACUC) of Shenzhen Institutes of Advanced Technology (approval number: SIAT-IACUC-20250912-YYS-NLDXZX-HC-A2387-01). For our experiments, 8–10 wk female C57Bl/6J mice (weight: 20–23 g, WT) and male C57BL/6J mice were purchased from the Beijing Vital River Laboratory Animal Technology Co., Ltd., China. These mice were housed under controlled 12 h light/dark cycles (temperature 20–22 °C; humidity 30–70%). We mated female C57BL/6J mice with male C57BL/6J mice and recorded the day of a copulatory plug as pregnancy day 0.5. At different embryonic days, C57BL/6J pregnant mice (total pregnant mice *n* = 25; E8.5, *n* = 4; E10.5, *n* = 8; E12.5, *n* = 6; E15.5, *n* = 4; E18.5, *n* = 3) were anesthetized with isoflurane and sacrificed with cervical dislocation to collect placental tissues.

For inhibitor-treated mice, E9.5 C57BL/6J pregnant mice were divided into two groups: (1) saline group (mice *n* = 4): E9.5 pregnancy mice were administrated with saline and a DMSO (10 μL) mixed solution (200 μL) through intraperitoneal injection; (2) inhibitor-treated group (mice *n* = 4): E9.5 pregnant mice were administrated with saline and a PD98059 solution (6 mg/kg) through intraperitoneal injection. The mice in the two groups were housed until E10.5, anesthetized with isoflurane, and sacrificed with cervical dislocation to collect placental tissues.

**Figure 7:**
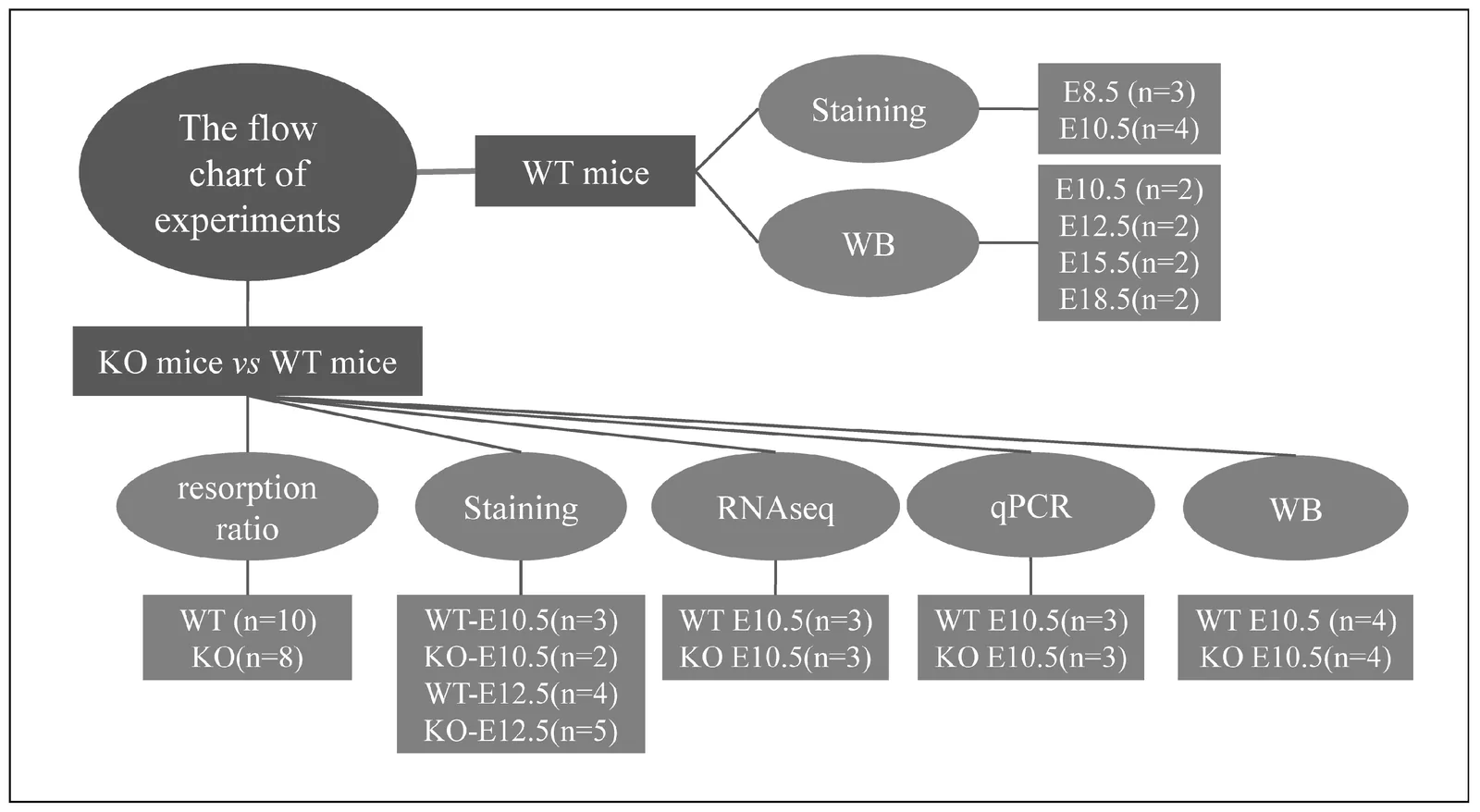
Flowchart of the experimental design.

AKR1B10 knockout mice with a C57BL/6J genetic background used in this study were purchased from Cyagen Bioscience and generated by targeting Exons 2 and 9 of murine AKR1B10 with a CRISPR-Cas9 sgRNA strategy. The sgRNA sequences were gRNA-A1: CAGATCCACGTGGACGCTGCTGG, gRNA-A2: CCTACAATGTGCTATGGCCAGGA; gRNA-B1: CCAAAGTGTGTTTTCCCCCAACC, gRNA-B2: CCTGGGGAATCACTAAGAGCAGA. These sgRNAs and Cas9 were microinjected into fertilized eggs to create the knockout mouse. The AKR1B10 KO genotype was determined from the PCR product. When the mice were pregnant, AKR1B10 knockout (KO) pregnant mice (total mice *n* = 15) were anesthetized with isoflurane and sacrificed at E10.5 (mice, *n* = 10) and E12.5 (mice, *n* = 5) to collect the placentas. The flow chart of the experimental design for WT and AKR1B10 KO mice is shown in Figure 7.

### 4.2. Ethics Approval for Human Placental Villi

Human placenta tissue experiments were approved by the Institutional Review Board of Shenzhen Institutes of Advanced Technology, Chinese Academy of Sciences (SIAT-IRB-180215-H0194) and the Shenzhen Nanshan District People’s Hospital Ethics Committee (No. 072652). In this study, we collected human placental villi from Shenzhen Nanshan District People’s Hospital. The placentas were obtained from pregnant women in the first trimester (6–10 weeks of gestation). These pregnant patients had no history of miscarriage, had undergone legal, voluntary terminations of early pregnancy, and signed informed consent forms to donate their placentas. The collected placenta villi were washed with cold PBS and fixed in 4% paraformaldehyde (PFA) for frozen sectioning.

### 4.3. Staining

The collected placenta tissues from mice and humans were fixed overnight at 4 °C in 4% paraformaldehyde (PFA). For tissue analysis, fixed tissues were dehydrated through a graded alcohol series and embedded with paraffin. Five-micron sections were used for hematoxylin and eosin (H&E) staining. For immunostaining, placental tissues were fixed with 4% PFA, dehydrated in 10% and 30% sucrose solutions, and subsequently embedded in optimal cutting temperature (OCT) compound. Placental tissues were sectioned at 10 μm thickness and incubated with 1% bovine serum albumin (Beyotime, ST023) after removing the OCT with PBS. The sections were incubated overnight with primary antibodies and the corresponding secondary antibodies. The antibodies in this study included primary antibodies (anti-AKR1B10 (ab96417, 1:200, Abcam, Shanghai, China), anti-cytokeratin 8 (CK8, TROMA-I, 1:200, DSHB, Iowa City, IA, USA), α-SMA (ab7817, 1:400, Abcam, Shanghai, China), and HLA-G (sc-21799, 1:200, Santa Cruz Biotechnology, Shenzhen, China)) and secondary antibodies (Goat anti-Rat IgG-Alexa Fluor™ 568 (A11077, 1:400, Thermo Scientific, Shanghai, China) and Donkey anti-Rabbit IgG-Alexa Fluor™ 488 (A21206,1:400, Thermo Scientific, Shanghai, China)). The tissue sections were placed under a coverslip with a mounting medium containing DAPI and examined under confocal laser scanning microscopy (TCS SP8, Leica, Hamburg, Germany).

The embedded human placentas in paraffin were twice dewaxed in a xylene solution for 10 min and rehydrated through decreasing concentrations of ethanol (100%, 90%, 80%, 70%, and 50% ethanol solution) for 5 min for immunohistochemical staining. The sections were washed with PBS and incubated with 5% bovine serum albumin (BSA; Beyotime, ST023, Shanghai, China). After blocking with BSA for 1 h, the sections were incubated with the primary antibody against AKR1B10 (18252-1-AP; 1:200) and the secondary antibody. The signal in the sections was visualized through the DAB substrate kit (Beyotime, P0203, Shanghai, China) for peroxidase detection and counterstained with hematoxylin. The images were then generated using an Olympus microscope (Olympus, BX53, Tokyo, Japan).

Periodic acid-Schiff (PAS) staining was conducted in the paraffin-embedded mouse placental sections. After dewaxing in xylene and rehydrating in a graded alcohol, tissue sections were oxidized in a periodic acid solution and then incubated in Schiff’s reagent for 15 min. Subsequently, sections were counterstained with hematoxylin, rinsed in Scott’s tap water, dehydrated through an alcohol gradient, and mounted with Entellan medium (C0142S, Beyotime, Shanghai, China).

### 4.4. The Spatiotemporal Transcript Expression of AKR1B10 in the Placenta

The spatial expression data for AKR1B10 in placental sections were generated from published stereo-seq datasets of E8.5 (E8.5_S1.MPSTA.h5ad, E8.5_S2.MPSTA.h5ad) and E10.5 (E10.5_S1.MPSTA.h5ad, E10.5_S2.MPSTA.h5ad), which were downloaded from the interactive data portal URL: https://db.cngb.org/stomics/mpsta/, accessed on 13 June 2025 [31], and a boxplot was generated from E8.5 and E10.5 placental sections.

### 4.5. RNA-Seq Analysis: Gene Expression and Analysis

RNA sequencing (RNA-seq) was performed on E10.5 placental tissues from wild-type (WT) and AKR1B10 knockout (KO) mice (*n* = 3 per group). Total RNA was extracted using the TRIzol reagent (15596026CN, Thermo Scientific, Shanghai, China), and its purity and integrity were assessed using a NanoDrop 2000 spectrophotometer (Thermo Scientific, Shanghai, China) and an Agilent 2100 Bioanalyzer (Agilent Technologies, Santa Clara, CA, USA). DNA sequencing libraries were constructed and sequenced on an Illumina NovaSeq 6000 platform by OE Biotech, Inc. (Shanghai, China). Transcript abundance was quantified as reads per kilobase per million mapped reads (RPKMs). Differential expression analysis was conducted with a false discovery rate (FDR) threshold of 0.05, and statistical significance was assessed using an empirical analysis of digital gene expression followed by Bonferroni’s correction. Subsequent Gene Ontology (GO) and GSEA analyses were performed using the OECloud online tool (https://cloud.oebiotech.com/task/, accessed on 20 October 2025). A *p*-value of <0.05 was considered statistically significant for enrichment results.

### 4.6. Quantitative Real-Time PCR

Total RNA was extracted from E10.5 placentas for WT (*n* = 5) and AKR1B10 KO (*n* = 5) mice using the RNAiso Plus reagent (Takara, Shiga, Japan) and analyzed by quantitative real-time PCR (qRT-PCR) according to the manufacturer’s instructions (Revert RT Master and ReverTaqPCR RT Master Mix gDNA remover, Toyobo, Osaka, Japan). These gene expressions were analyzed in placental RNAs, with primer sequences shown in Table 1. Gene expressions in placental tissue were normalized to 18S and GAPDH using the ΔΔCT method, and the relative expression level was calculated for the WT group. Melt curve analyses for each primer set showed a single peak for each product.

### 4.7. AKR1B10 shRNA Lentiviral Vector Production

To construct lentiviral vectors for AKR1B10 knockdown, the target sequence (ATGGCCACGTTTGTGGAGCTCAGTACCAAAGCCAAGATGCCCATTGTGGGCCTGGGCACTTGGAAGTCTC) and a non-targeting scramble control sequence (CCTAAGGTTAAGTCGCCCTCG) were cloned into the pLV[shRNA]-EGFP:T2A: Puro-U6 vector (Cyagen, Suzhou, China). The recombinant plasmids were transfected into HEK293T packaging cells. Lentivirus-containing supernatants were collected at 48 h post-transfection, and the viral titer was quantified using the QuickTiter Lentivirus Quantitation Kit (Cell Biolabs, Shanghai, China). HTR-8/SVneo cells were subsequently infected with the harvested lentivirus to generate stable AKR1B10 knockdown and control cell lines. The efficiency of AKR1B10 knockdown was confirmed at the protein level.

### 4.8. Cell Line Culture and Wound Healing Assay

HTR-8/SVneo cell lines stably infected with shAKR1B10 or scrambled shRNA (shNC) were cultured in a 1:1 mixture of Dulbecco’s Modified Eagle Medium (DMEM; HyClone, Cytiva, Shanghai, China) supplemented with 10% fetal bovine serum (FBS; Thermo Scientific, Shanghai, China), 100 U/mL penicillin, and 100 μg/mL streptomycin, and they were maintained at 37 °C in a humidified 5% CO_2_ incubator. For the wound healing assay, cells were seeded into 24-well plates and grown to confluence. After overnight serum starvation, a linear scratch was made in each well using a sterile 200 μL pipette tip. Cells were then allowed to migrate for 0, 12, and 24 h in the serum-free medium. At each time point, eight randomly selected fields per well were imaged under an Olympus BX53 microscope. The migratory area was quantified using ImageJ software version 1.54 by measuring the change in wound width: Migration distance = (Width at 0 h) − (Width at T h). Data are presented as mean ± SEM from independent experiments, where each experiment included three replicate wells per group.

### 4.9. Transwell Assay

HTR8/SVneo cells were harvested with 0.25% trypsin-EDTA and resuspended in a serum-free medium at a concentration of 2 × 10^5^ cells/mL. The cell suspension (about 100 μL) was added to the upper chamber of the transwell insert (Corning, Shanghai, China). The lower chamber was filled with 600 μL of the complete medium. The plates were incubated at 37 °C in a humidified atmosphere with 5% CO_2_ for 24 h. After incubation, the cells on the upper surface of the membrane were carefully removed using a cotton swab. The migrated cells on the lower surface were fixed with 4% paraformaldehyde and stained with 0.1% crystal violet solution. The chambers were washed gently with PBS to remove excess dye, air-dried, and visualized under a microscope (Olympus BX53, Japan). Cells in five random fields of view were counted for each well to determine the average number of migrated cells.

### 4.10. Western Blot Assay

Total protein was extracted from HTR8/SVneo cells and placental tissues using a lysis buffer (Thermo Scientific, 78501, Shanghai, China) and quantified with the Pierce BCA Protein Assay Kit. Equal amounts of protein were separated by SDS-PAGE and transferred onto polyvinylidene fluoride (PVDF) membranes. After blocking with 5% non-fat milk, the membranes were incubated overnight at 4 °C with the following primary antibodies: anti-AKR1B10 (18252-1-AP; Poteintech, Wuhan, China), anti-Erk1/2 (4695S; Cell Signal, Shanghai, China), anti-phospho-p44/42 Erk1/2 (Thr202/Tyr204) (4370S; Cell Signal, Shanghai, China), anti-PPARγ (#2435; Cell Signal, Shanghai, China), anti-CD36 (18836-1-AP; Poteintech, Wuhan, China), anti-FABP4 (12802-1-AP, Poteintech, Wuhan, China), anti-FASN (10624-2-AP, Poteintech, Wuhan, China), anti-ACC (3676s, Cell Signal, Shanghai, China), anti-ACTN (#4970; Cell Signal, Shanghai, China), and anti-α-tubulin (10094-1-AP; Poteintech, Wuhan, China). Following washing with TBST, membranes were incubated with horseradish peroxidase-conjugated secondary antibodies at room temperature for 1 h. Protein bands were visualized using an enhanced chemiluminescence substrate (4AW011-100, 4A Biotech Co., Ltd., Beijing, China). Three repeated protein expressions were measured with ImageJ, and the relative protein level was calculated according to the tubulin and ACTN expression.

### 4.11. Statistical Analyses

Statistical analyses were conducted with GraphPad Prism 7. Normality was determined via Shapiro–Wilk tests and Q–Q plots, and variance equality was checked using the F-test. Normally distributed data were compared using two-tailed unpaired Welch’s *t*-test, while non-normally distributed data were analyzed with a non-parametric Mann–Whitney U test. Data are shown as mean ± SD. The significance levels are as follows: * *p* < 0.05, ** *p* < 0.01, and *** *p* < 0.001.

## Figures and Tables

**Table 1:** The sequences of primers.

Gene Name	NM-Number	Primers Sequences	Product Size (bp)	Melting Temperature (°C)
*AKR1B10*	NM_172398	F: 5-CCTCAAGGACACGGAACCAG-3, R: 5-TTAGCTGGGGGAGACTTCCA-3	165	84
*Tpbpα*	NM_009411	F: 5-TTCCTAGTCATCCTATGCCTGG-3, R: 5-TGGTCATTTTCGCTACTGTGAAG-3	238	82
*Hand1*	NM_008213	F: 5-GGGTTAAACCCGGTCTTTGG-3, R: 5-AAGGACCTGCCGACCTCTTG-3	79	82
*ASCL2*	NM_008554	F: 5-ACCTACCTGCTTCTAGCCCA-3, R: 5-CACTTGGCATTTGGTCAGGC-3	80	82
*Prl7b1*	NM_029355	F: 5-GGGAGGACGTGGTCTCTGTA-3, R: 5-TTTGGTGATTTGAGTGGCAA-3	256	81
*Synα*	NM_001013751	F: 5-CCTCACCTCCCAGGCCCCTC-3, R: 5-GGCAGGGAGTTTGCCCACGA-3	82	82
*Eomes*	NM_001164789	F: 5-GGCCCCTATGGCTCAAATTCC-3, R: 5-GAACCACTTCCACGAAAACATTG-3	141	82
*Cdx2*	NM_007673	F: 5-GTTCTGTCCCTGGGGTTCTG-3, R: 5-TTCTCGCAGCGTCCATACTC-3	137	86
*AKR1B7*	NM_009731	F: 5-CAGGGATTTCAGGCTGGGAA-3, R: 5-GGGTGGCTCTCAATCTGGTT-3	227	84
*18s*	NR_003278	F: 5-CAGAGAGTGGCGATGGGTTTT-3, R: 5-GACAATGGCACAGTGGCTGTT-3	128	79
*Gapdh*	NM_001289726	F: 5-AGGTCGGTGTGAACGGATTTG-3, R: 5-TGTAGACCATGTAGTTGAGGTCA-3	123	84

## Data Availability

The original contributions presented in this study are included in the article and Supplementary Materials. Further inquiries can be directed to the corresponding authors.

## Supplementary Materials

The following supporting information can be downloaded at https://www.mdpi.com/article/10.3390/ijms27177728/s1.
